# A study protocol for a feasibility study: Propofol Target-Controlled Infusion in Emergency Department Sedation (ProTEDS)—a multi-centre feasibility study protocol

**DOI:** 10.1186/s40814-019-0412-y

**Published:** 2019-02-18

**Authors:** Fiona M. Burton, David J. Lowe, Jonathan Millar, Alasdair R. Corfield, Malcolm A. B. Sim

**Affiliations:** 1 Department of Emergency Medicine, University Hospital Hairmyres, Eaglesham Road, Glasgow, G75 8RG UK; 2 Glasgow University Section of Anaesthesia, Pain and Critical Care, Glasgow, Scotland; 3 Department of Emergency Medicine, Queen Elizabeth University Hospital, Glasgow, Scotland; 4 Department of Anaesthesia and Critical Care, Queen Elizabeth University Hospital, Glasgow, Scotland; 5 Department of Emergency Medicine, Royal Alexandra Hospital, Paisley, Scotland

**Keywords:** Target-controlled infusion, Propofol, Procedural sedation, Adverse events

## Abstract

**Background:**

Procedural sedation is a core skill of the emergency physician. Bolus administration of propofol is widely utilised in UK emergency departments to provide procedural sedation. Bolus administration of propofol, titrated to an endpoint of sedation, has a rapid effect but can easily result in apnoea and loss of airway patency. The use of a target-controlled infusion of propofol allows for controlled titration to an effect site concentration and may reduce the rate of adverse incidents. Target-controlled infusion of propofol is not currently used in emergency departments.

The primary aim of this feasibility study is to ensure that propofol target-controlled infusion (TCI) is acceptable to the patient and that recruitment rates are adequate to power a randomised controlled trial comparing propofol target-controlled infusion versus bolus administration.

**Methods:**

This study will recruit in four emergency departments in Scotland, UK. Patients aged 18–65 years with anterior shoulder dislocation, weighing ≥ 50 kg and fasted ≥ 90 min, will be screened. Recruited patients will undergo emergency reduction of a dislocated shoulder facilitated by procedural sedation utilising TCI of propofol.

The widespread adoption of TCI propofol by emergency departments will require evidence that it is safe, potentially effective, patient centred and a timely method of providing procedural sedation. The primary endpoint will be acceptability measured by patient satisfaction. The secondary endpoints will include incidence and severity of adverse events, number of shoulder reduction attempts, nursing opinion of patient experience, patient’s reported pain score and time from commencement of TCI propofol sedation to desired sedation level.

The study will be open for recruitment from April 2017 to December 2018.

**Discussion:**

If the study demonstrates patient acceptability with adequate recruitment, we will be in a position to determine the feasibility of progression to a randomised controlled clinical trial of TCI compared to bolus administration of propofol.

**Trial registration:**

ClinicalTrials.gov Identifier: NCT03442803. Registered retrospectively on 22 February 2018.

**Electronic supplementary material:**

The online version of this article (10.1186/s40814-019-0412-y) contains supplementary material, which is available to authorized users.

## Background

### Sedation in the emergency department

Procedural sedation and analgesia (PSA) has long been a core skill of the emergency physician, although over the last decade, developments in patient monitoring and the use of newer sedative and analgesic agents have served to improve both safety and efficacy [1]. Despite these advances and their consolidation into well-designed guidelines [2, 3], concern regarding the safety of emergency department (ED) PSA persists [4, 5].

More specifically, concerns have been raised regarding the use of propofol, an ultra-short acting anaesthetic agent [6]. The practice of using sub-anaesthetic doses of propofol to achieve sedation in ED PSA originated around the turn of the millennium [7] and has since become the most common choice of sedative in the ED [4, 8]. Propofol offers a number of advantages as a sedative agent, including a short onset and recovery time, amnesiac properties and good efficacy [9–11]. The cost of these properties is, in part, a narrow therapeutic range. Propofol in the ED is currently given as a repeated bolus (normally a few millilitres at a time until the desired effect is achieved).

When given as a bolus, propofol will induce a spectrum of states up to and including general anaesthesia, dependent on the dosage. The correlation between dose and effect varies based on several patient factors. Whilst targeting a state of sedation, it is possible for the operator to ‘overshoot’ moving rapidly from conscious sedation to deep sedation to general anaesthesia. The inadvertent induction of general anaesthesia may result in unanticipated complication. The principle complications associated with accidental over-sedation or general anaesthesia relate to the patency of the patient’s airway. The reported frequency of airway complications during ED PSA with propofol range from 5.0 to 9.4% [7]; this includes a rate of supplemental ventilation of between 3.0 and 9.4%, with oxygen desaturation occurring in between 5 and 7% of cases [12]. In addition, the bolus administration of propofol may result in transient hypotension [12]. Despite being a relatively short-lived effect, this may be pronounced in those with intravascular volume depletion [13] or in the elderly [14], and in some may be profound, with one series demonstrating that 3.5% of those undergoing PSA with propofol experience ≥ 20% falls in blood pressure [15]. At first inspection, the reported rate of complication may appear low and has by some been interpreted as evidence of the relative safety of ED PSA; however, alternative views have been expressed in the context of much lower rates of adverse events seen in elective painful procedures requiring conscious sedation [6, 16]. This is compounded by the non-standard way in which adverse events have been reported across studies [17].

A potential solution to the adverse events experienced with the bolus administration of propofol is the use of a target-controlled infusion (TCI). This method is widely used in anaesthetic practice, and whilst the PK remains the same irrespective of if you give a bolus or infusion of propofol, TCI allows the titration in perhaps a more controlled manner.

### Target-controlled infusion

The aim when administering propofol, as with any drug, is to induce a desired clinical effect, in this context a level of sedation. As previously alluded to, when administering propofol in a bolus fashion or as a fixed rate infusion, without regard to those factors which cause biologic variability (e.g. age, gender, weight), it is difficult to accurately and consistently predict clinical effect. The development, in the early 1990s, of computer-assisted infusion devices, for the first time, allowed the clinician to target a plasma concentration, with the pump automatically altering the rate of infusion based on a pre-programmed pharmacokinetic model [18]. These target-controlled infusion (TCI) devices have undergone significant development and are now in widespread clinical use. Their operation uses a mathematical mode that reflects a theoretical ‘three-compartment’ model that may comprise the central compartment (plasma), and two peripheral compartments (highly perfused tissue, e.g. brain, and poorly perfused tissue, e.g. adipose). In a state of equilibrium, propofol will diffuse between compartments at a constant rate. These rate constants have been used in pharmacokinetic models to mathematically predict the plasma concentration and latterly the effect site concentration, in this case that in the brain [19].

In practical terms, TCI allows the operator to more accurately target a specific clinical effect. When propofol is administered as a bolus, the operator is likely to either underdose, delivering an insufficient effect site concentration, or overdose, exceeding the desired effect site concentration. TCI allows the operator to titrate to effect and then to maintain a steady state, potentially eliminating the risk of ‘over shooting’ and reducing the rate of adverse events. TCI is not without limitation. PK models are an estimate as they have been derived from a healthy population, but any inherent inaccuracy is consistent in different populations and accounted for by careful titration.

The use of propofol TCI has been studied in a number of settings, including gastrointestinal endoscopy [20–22], dental surgery [23, 24], oocyte retrieval [6] and bronchoscopy [25, 26]. To our knowledge, propofol TCI in sedation has not been studied in an ED setting. Trials have demonstrated a good safety profile for propofol TCI, with at least one large randomised controlled trial [20] in an endoscopy setting, showing a reduction in both respiratory and cardiovascular adverse events in comparison to the bolus administration of propofol. Unfortunately, trials to date have suffered from a high degree of heterogeneity, leading the Cochrane review, on the subject of propofol TCI versus manually controlled infusion in both general anaesthesia and sedation, to conclude that there was insufficient evidence to make firm recommendations regarding its use in clinical practice [27].

### Study rationale

There exists continued controversy over the use of propofol in ED PSA; this is despite its widespread existence in clinical practice for at least a decade. These concerns are not limited to the ED setting and are primarily related to the pharmacological properties of the drug itself and its potential for harm. The bolus administration of propofol, aimed at a target of sedation, offers several advantages over more traditional agents, yet these advantages are also its limitations. The use of a target-controlled infusion makes titration of propofol easier with fewer adjustments, thus potentially reducing the incidence of adverse incidents.

The incidence of adverse events using bolus propofol versus TCI propofol in the emergency department will be recorded in the future randomised controlled trial (RCT). This current feasibility study is needed first before a main trial in order to provide evidence that TCI is safe, potentially effective, patient centred and a timely method of providing procedural sedation. It will also provide information about recruitment to ensure that the randomised controlled trial can be adequately powered.

### Aim

The primary aim of this feasibility study is to ensure that propofol TCI is acceptable to the patient and that recruitment rates are adequate to power a future RCT.

Secondary objectives are:

- Safety:
  - ◦ Incidence of adverse events
- Potential effectiveness:
  - ◦ Successful completion of the procedure
  - ◦ Number of reduction attempts
- Patient centred:
  - ◦ Patient-reported pain scores
  - ◦ Nursing opinion of the patient’s experience
  - ◦ Patient recall of the procedure
  - ◦ Free text comments from all staff at the end of the procedure
- Timely:
  - ◦ Time taken to reach the appropriate sedation level
  - ◦ Time from commencement of sedation to ‘fit for discharge’

### Progression

Progression to a multi-centre randomised control trial will require evidence of the ability to adequately recruit and that it is safe, potentially effective, patient centred and a timely method of providing procedural sedation.

## Methods

### Study design

Multi-centre feasibility study

### Participating centres

- University Hospital Hairmyres ED, UK
- Royal Alexandra Hospital ED, UK
- Glasgow Royal Infirmary ED, UK
- Queen Elizabeth University Hospital ED, UK

### Study population

Adult patients (≥ 18 years), requiring sedation to facilitate the reduction of an acute traumatic anterior shoulder dislocation in the emergency department

### Inclusion criteria

- Aged 18–65 years
- Clinical and/or radiological evidence of acute anterior shoulder dislocation
- American Society of Anesthesiologists (ASA) Physical Status Classification I or II
- Fasted ≥ 90 min [2, 3, 28, 29]
- Weight ≥ 50 kg

### Exclusion criteria

- Inability to provide or refusal of informed consent
- Previous attempt at reduction during the same presentation
- Previously enrolled in the study
- Clinical and/or radiological evidence of acute posterior shoulder dislocation
- Clinical and/or radiological evidence of concomitant ipsilateral upper limb fracture (with the exception of an isolated avulsion fracture of the greater tuberosity or a fracture of the glenoid labrum)
- Concomitant multi-system injury
- History of difficult intubation/airway surgery
- ASA grades III, IV or V
- Haemodynamic instability
- Pregnancy
- Contraindication to sedation
- Allergy to study drugs or eggs
- Clinician decision
- Morphine administration within the preceding 20 min prior to starting TCI (can be included if > 20 min)

There is no objection to subsequent co-enrolment of patients to clinical trials amongst those already enrolled to ProTEDS.

### Sample size rationale

A formal sample size should not be calculated for this feasibility study [30]. We aim to recruit at least 20 patients within a fixed time period to allow calculation of recruitment rate. This time period was agreed by reviewing the average number of anterior shoulder dislocations attending at each site per week.

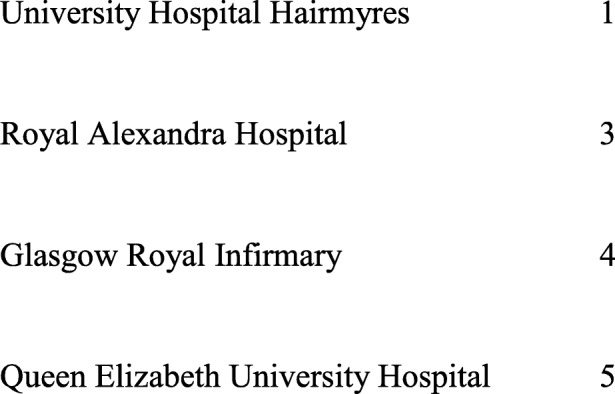



Recruitment commenced on April 2017 and will continue until 31 December 2018. If 13 patients per week present for 86 weeks of the planned duration of the study, then a total of 1118 potential patients could potentially be recruited. After we account for those patients who are willing and able to give consent (approximately 1 in 5) to research in an emergency care setting (1118/5), we estimate that 224 potential patients could be recruited. A total of one in five of the consultant staff at the recruiting sites was trained to undertake this procedure using TCI propofol (224/5) leaving 45 patients presenting at any time during a 24-h period. The consultant presence in the emergency department is approximately 12 h per 24 h (45/2), leaving approximately 22 patients from the total of 1116 anterior shoulder dislocations.

This information will allow assessment of the feasibility of recruiting to an adequately powered randomised controlled trial with the incidence of adverse events as the primary endpoint.

Emergency departments have a unique set of challenges that make recruiting to research studies difficult [31]. Patients may present at any point, 24 h per day, 7 days a week to an unpredictable, high-risk setting. All research activity is undertaken by emergency medicine consultants.

### Primary endpoints

- Patient satisfaction—visual analogue scale (VAS) (Additional file 1) [32]
- Percentage of patients recruited vs percentage of patients approached

### Secondary endpoints

- Incidence and severity of adverse events per World Society for Intravenous Anaesthesia (SIVA) adverse event sedation reporting tool [33]
- The successful reduction of the anterior shoulder dislocation
- Number of reduction attempts
- Patient-reported pain score VAS (Additional file 2) [32, 34]
- Nurse opinion of patient experience VAS (Additional file 3) [32]
- Patient recall of the procedure [35]
- Free text comments from all staff at the end of the procedure
- Time from commencement of induction to Modified Observer’s Assessment of Alertness/Sedation Scale (OAA/S) 3 [36]
- Time from commencement of sedation to fit for discharge [2]
  - ◦ Patient returned to their baseline level of consciousness
  - ◦ Vital signs are within normal limits for that patient
  - ◦ Respiratory status is not compromised
  - ◦ Pain and discomfort have been addressed

**Fig. 1:**
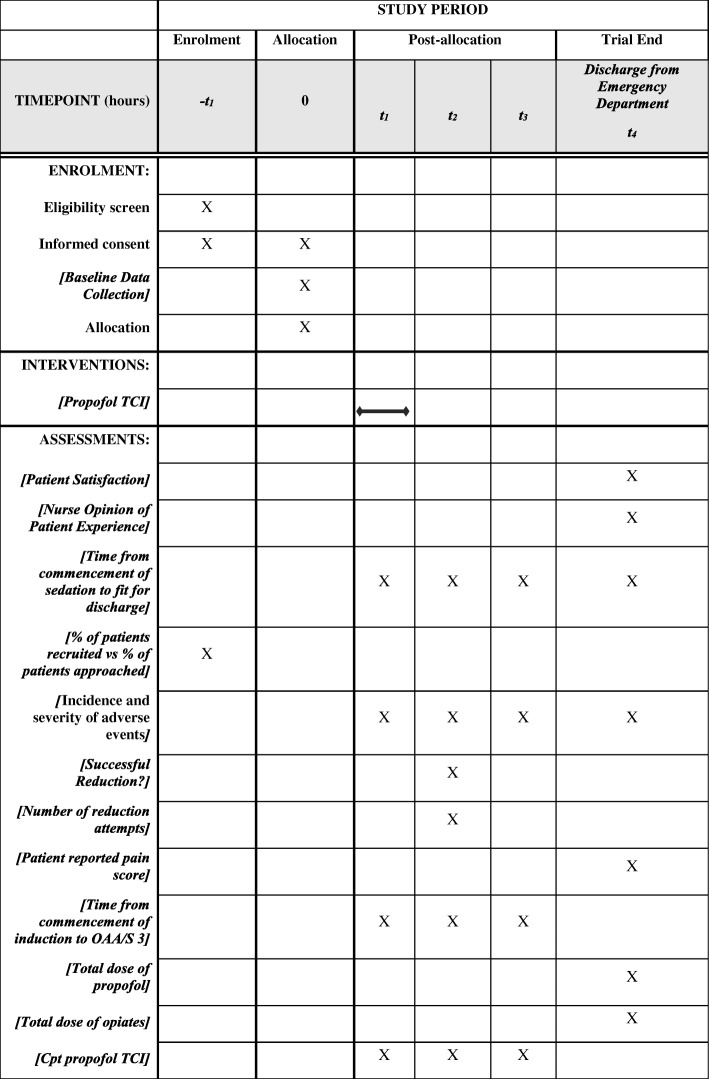
Standard Protocol Items: Recommendations for Interventional Trials (SPIRIT) figure of enrolment, interventions and assessments

Figure 1 displays the Standard Protocol Items: Recommendations for Interventional Trials (SPIRIT) figure of enrolment, interventions and assessments.

### Study procedures

Recruiting clinicians will be consultants in emergency medicine who currently use bolus propofol as sedation. They have a valid Good Clinical Practice Certificate. They are responsible for obtaining consent from patients and delivering the TCI propofol as per the flowsheet (Additional file 4). The clinicians have received aditional training in TCI propofol by an experienced anaesthetist (author MS). The recruiting clinician will be solely responsible for the administration of TCI propofol but not in the reduction of the anterior shoulder dislocation.

Potential participants presenting to the emergency department with a suspected shoulder dislocation will receive a patient information sheet in addition to their standard clinical care. On confirmation of a shoulder dislocation, they will be screened against the inclusion criteria by clinical staff within participating emergency departments.

Where a patient satisfies these criteria, the trained clinician, if on duty, will liaise with the clinical team to establish whether or not the patient is eligible for enrolment based on the formal inclusion and exclusion criteria detailed in the protocol.

In line with the current best practice [2], a minimum array of patient monitoring will be prescribed: 3-lead electrocardiogram, non-invasive blood pressure, peri pheral oxygen saturation and end-tidal carbon dioxide monitoring. There will be one clinician responsible for sedating the patient and another clinician undertaking the reduction. The sedating clinician will be assisted by a skilled assistant, normally an emergency department nurse. This is already undertaken as routine in all patients undergoing procedural sedation [2]. In addition, all patients will receive supplemental oxygen (via nasal cannulae at 4 L min^−1^) for the duration of the sedation episode.

Following patient enrolment, the TCI sedation flow sheet (Additional file 4) will be followed. The patient can receive morphine analgesia as long as it is administered at least 20 min before commencement of sedation. The dose and time of administration of morphine will be recorded.

The patient’s physiological parameters including heart rate, blood pressure and end-tidal carbon dioxide levels will be recorded prior to the administration of sedation and every 3 min until 15 min post reduction; thereafter, these will be recorded on a 15-min basis until full recovery. Likewise, the peripheral oxygen saturation will be recorded except with increased frequency, at 1-min intervals until 15 min post reduction upon which time they will be recorded on a 15-min basis. The non-invasive blood pressure will be obtained by measuring the posterior tibial pressure as we will be unable to use either arm owing to the dislocation and the TCI. When the patient’s modified observer’s assessment of alertness/sedation score (Additional file 5) reaches the target of 3, it will be recorded every 3 min until the procedure is complete. A patient-reported pain score for the procedure will be obtained after full recovery (Additional file 3).

Patients will be consented for the procedure and inclusion in the study by the responsible clinician. Written consent for use of the patient’s information and administering the propofol via TCI route as opposed to the standard bolus administration will be obtained pre-procedure.

Written consent will be sought from the patient to allow usage of their data collected during their stay in the ED and for administering the propofol via the TCI route. The procedure to relocate the shoulder and sedative agent being used would be the same regardless of their inclusion or exclusion in the study; it is only the method of administering the propofol that differs. The patient will be asked to sign the patient consent form that will then be counter signed by the responsible clinician. The patient will retain one copy of the signed consent form, and a further copy will be placed in the patient’s medical records whilst the original will be retained by the principal investigator.

The right of the participant to refuse to participate without giving reasons must and will be respected. All participants are free to withdraw at any time from the study without giving reasons and without prejudicing further treatment. Any data collected that is additional to the routine will be destroyed.

For each patient, a number of demographic, clinical and physiological parameters will be collected via clinical information systems and previous healthcare records.

### Data collection and management

A standardised data collection sheet will be utilised to record information for each patient enrolled in the study. The data collection sheet will be our source document. All data entered on the data collection sheet is hand written, and the information transcribed from a screen. On completion of the data collection sheet, they will be signed and dated. These will be maintained in an investigator file to be secured in a locked office within the study site. Information recorded on the data collection sheet will be recorded in a database located on a secure server.

Required data, other than that recorded at the time of sedation, will be retrieved from existing clinical IT systems and by review of the patient’s hospital notes. It is the principal investigators responsibility to ensure that all data is handled in a confidential fashion and in compliance with the protocol, local policy and statutory requirements.

The following is a non-exhaustive list of variables that will be recorded for each enrolled patient:

- Age
- Gender
- ED length of stay
- ED discharge destination
- Mechanism of shoulder dislocation
- Number of previous presentations following anterior shoulder dislocation
- Pre-sedation physiology
- Time of verbal and written consent

### Planned analysis

The plan of analysis for study data will be designed with the assistance of a biomedical statistician from the University of Strathclyde. Descriptive statistics will be presented.

Following the completion of this feasibility study, we plan to proceed to an RCT. The primary endpoint for our multi-centre RCT will be the incidence and severity of adverse events.

### Ethical approval and amendments

Ethical and amendment approval were obtained from the West of Scotland Research Ethics Committee 5, reference number 17/WS/0020 on 24 January 2017.

### Patient confidentiality

The principal investigator will preserve the confidentiality of participants taking part in the study in line with the Data Protection Act. Data held centrally by the principal investigator will be anonymised were practical and identified by a unique code. Documents that are not anonymised, such as signed informed consent forms, will be kept in a strictly confidential file by the principal investigator.

### Adverse event reporting

A serious adverse event occurring to a research participant will be reported to the main REC within 15 days of the chief investigator, or designee becoming aware where in the opinion of the chief investigator the event was related and unexpected. They will be reported as outlined in the ‘Safety and Progress Reports Table (non-CTIMPs) for UK health departments’ RES version 2.1 05.06.2015’.

### Protocol deviation reporting

A protocol deviation is any departure from the approved protocol. All deviations will be recorded and reported to the sponsor. Prospective protocol deviations will not be authorised by the sponsor unless the deviation is necessary to eliminate an immediate hazard.

### Publication policy

All publications and presentations relating to the study will be authorised by the chief investigator. Authorship will be determined according to the internationally agreed criteria for authorship (www.icmje.org). Our sponsor will review all documents prior to publication.

## Discussion

Research in emergency medicine is challenging owing to the unpredictable nature of the specialty and paucity of research support [31]. In the context of this challenging environment, a decision will be taken as to whether recruitment is adequate to allow progression to a randomised controlled trial. All observational studies have potential bias, and the results should generally not be used to change practice. The results of observational studies can be used to inform the development protocols for randomised clinical trials. The purpose of this study is to determine whether TCI propofol has the potential in terms of patient satisfaction to offer an alternative method for procedural sedation in the emergency department. The chosen primary endpoint of patient satisfaction scores will be inherently less prone to bias as patients are unlikely to have a bias for procedural sedation. In order to progress to a randomised control trial of TCI propofol against bolus propofol, TCI propofol would have to be no worse than standard bolus propofol in terms of patient satisfaction and also offer the potential of improved patient safety to be an acceptable clinical alternative for procedural sedation.

The purpose of the future multi-centre randomised trial is to compare the safety of propofol sedation, delivered via a target-controlled infusion, in comparison to that of usual bolus administration for the relocation of acute traumatic shoulder dislocation in adult patients in the emergency department. Our primary endpoint for that RCT will be the incidence of adverse events as recorded on the World SIVA adverse event sedation reporting tool [33]. We believe that the results will be generalisable to other painful procedures in the emergency department.

## Trial status

The trial is currently open for recruitment. The Standard Protocol Items Recommendations for Trials (SPIRIT) checklist has been added as Additional file 6.

## Additional files

Additional file 1: Patient satisfaction—visual analogue scale (VAS) (PDF 30 kb)

Additional file 2: Nurse opinion of patient experience VAS (PDF 24 kb)

Additional file 3: Patient-reported pain score VAS (PDF 27 kb)

Additional file 4: The TCI sedation flow sheet (PDF 71 kb)

Additional file 5: A patient’s modified observer’s assessment of alertness/sedation score (PDF 37 kb)

Additional file 6: The Standard Protocol Items Recommendations for Trials (SPIRIT) checklist (PDF 78 kb)

## Abbreviations

ASA: American Society of Anesthesiologists
ED: Emergency department
OAA/S: Observer’s Assessment of Alertness/Sedation Scale
PSA: Procedural sedation and analgesia
SIVA: Society for Intravenous Anaesthesia
TCI: Target-controlled infusion
VAS: Visual analogue scale

## References

[1] Green SM (2007). Research advances in procedural sedation and analgesia. Ann Emerg Med.

[2] The Royal College of Anaesthetists and The College of Emergency Medicine Working Party on Sedation (2012). Safe sedation of adults in the emergency department.

[3] Godwin SA, Burton JH, Gerardo CJ, Hatten BW, Mace SE, Silvers SM (2014). Clinical policy: procedural sedation and analgesia in the emergency department. Ann Emerg Med.

[4] Jacques KG, Dewar A, Gray A, Kerslake D, Leal A, Lees F (2011). Procedural sedation and analgesia in a large UK emergency department: factors associated with complications. Emerg Med J.

[5] Mathieu N, Jones L, Harris A, Hudson A, McLauchlan C, Riou P (2009). Is propofol a safe and effective sedative for relocating hip prostheses?. Emerg Med J.

[6] Edwards J, Kinsella J, Shaw A, Evans S, Anderson KJ (2010). Sedation for oocyte retrieval using target controlled infusion of propofol and incremental alfentanil delivered by non-anaesthetists. Anaesthesia.

[7] Miner JR, Krauss B (2007). Procedural sedation and analgesia research: state of the art. Acad Emerg Med.

[8] Bell A, Taylor DM, Holdgate A, MacBean C, Huynh T, Thom O (2011). Procedural sedation practices in Australian emergency departments. Emerg Med Australas.

[9] Hohl CM, Sadatsafavi M, Nosyk B, Anis AH (2008). Safety and clinical effectiveness of midazolam versus propofol for procedural sedation in the emergency department: a systematic review. Acad Emerg Med.

[10] Zed PJ, Abu-Laban RB, Chan WWY, Harrison DW (2007). Efficacy, safety and patient satisfaction of propofol for procedural sedation and analgesia in the emergency department: a prospective study. Can J Emerg Med.

[11] Miner JR, Danahy M, Moch A, Biros M (2007). Randomized clinical trial of etomidate versus propofol for procedural sedation in the emergency department. Ann Emerg Med.

[12] Miner JR, Burton JH (2007). Clinical practice advisory: emergency department procedural sedation with propofol. Ann Emerg Med.

[13] Illievich UM, Petricek W, Schramm W, Weindlmayr-Goettel M, Czech T, Spiss CK (1993). Electroencephalographic burst suppression by propofol infusion in humans: hemodynamic consequences. Anesth Analg.

[14] Kazama T, Ikeda K, Morita K, Kikura M, Doi M, Ikeda T (1999). Comparison of the effect-site keOs of propofol for blood pressure and EEG bispectral index in elderly and younger patients. J Am Soc Anesthesiol.

[15] Burton JH, Miner JR, Shipley ER, Strout TD, Becker C, Thode HC (2006). Propofol for emergency department procedural sedation and analgesia: a tale of three centers. Acad Emerg Med.

[16] Davison M, Stewart R (2009). Is propofol a safe and effective sedative for relocating hip prostheses?. Emerg Med J.

[17] Newstead B, Bradburn S, Appelboam A, Reuben A, Harris A, Hudson A (2013). Propofol for adult procedural sedation in a UK emergency department: safety profile in 1008 cases. Br J Anaesth.

[18] Goudra BG, Mandel JE (2009). Target-controlled infusions/patient-controlled sedation. Tech Gastrointest Endosc.

[19] Frölich M, Dennis D, Shuster J, Melker R (2005). Precision and bias of target controlled propofol infusion for sedation. Br J Anaesth.

[20] Chan W-H, Chang S-L, Lin C-S, Chen M-J, Fan S-Z (2014). Target-controlled infusion of propofol versus intermittent bolus of a sedative cocktail regimen in deep sedation for gastrointestinal endoscopy: comparison of cardiovascular and respiratory parameters. J Dig Dis.

[21] Gillham MJ, Hutchinson RC, Carter R, Kenny GNC (2001). Patient-maintained sedation for ERCP with a target-controlled infusion of propofol: a pilot study. Gastrointest Endosc.

[22] Eberl S, Preckel B, Bergman J, Hollmann M (2013). Safety and effectiveness using dexmedetomidine versus propofol TCI sedation during oesophagus interventions: a randomized trial. BMC Gastroenterol.

[23] Sakaguchi M, Higuchi H, Maeda S, Miyawaki T (2011). Dental sedation for patients with intellectual disability: a prospective study of manual control versus Bispectral Index-guided target-controlled infusion of propofol. J Clin Anesth.

[24] Oei-Lim V, Kalkman C, Makkes P, Ooms W (1998). Patient-controlled versus anesthesiologist-controlled conscious sedation with propofol for dental treatment in anxious patients. Anesth Analg.

[25] Lin T, Lo Y, Hsieh C, Ni Y, Wang T, Lin H (2013). The potential regimen of target-controlled infusion of propofol in flexible bronchoscopy sedation: a randomized controlled trial. PLoS One.

[26] Franzen D, Bratton DJ, Clarenbach CF, Freitag L, Kohler M (2016). Target-controlled versus fractionated propofol sedation in flexible bronchoscopy: a randomized noninferiority trial. Respirology.

[27] Leslie K, Clavisi O, Hargrove J (2016). Target-controlled infusion versus manually-controlled infusion of propofol for general anaesthesia or sedation in adults. Cochrane Database Syst Rev.

[28] Thorpe RJ, Benger J (2010). Pre-procedural fasting in emergency sedation. Emerg Med J.

[29] Kanji A, Atkinson P, Fraser J, Lewis D, Benjamin S (2016). Delays to initial reduction attempt are associated with higher failure rates in anterior shoulder dislocation: a retrospective analysis of factors affecting reduction failure. Emerg Med J.

[30] Dixon WJ (1965). The up-and-down method for small samples. J Am Stat Assoc.

[31] Cofield SS, Conwit R, Barsan W, Quinn J (2010). Recruitment and retention of patients into emergency medicine clinical trials. Acad Emerg Med.

[32] McCormack HM, David JL, Sheather S (1988). Clinical applications of visual analogue scales: a critical review. Psychol Med.

[33] Mason KP, Green SM, Piacevoli Q (2012). Adverse event reporting tool to standardize the reporting and tracking of adverse events during procedural sedation: a consensus document from the World SIVA International Sedation Task Force. Br J Anaesth.

[34] Breivik EK, Björnsson GA, Skovlund E (2000). A comparison of pain rating scales by sampling from clinical trial data. Clin J Pain.

[35] Pandit JJ, Andrade J, Bogod DG, Hitchman JM, Jonker WR, Lucas N (2014). 5th National Audit Project (NAP5) on accidental awareness during general anaesthesia: summary of main findings and risk factors. Br J Anaesth.

[36] Chernik DA, Gillings D, Laine H, Hendler J, Silver JM, Davidson AB (1990). Validity and reliability of the Observer’s Assessment of Alertness/Sedation Scale: study with intravenous midazolam. J Clin Psychopharmacol.

