# Supplementary material for: A study protocol for a feasibility study: Propofol Target-Controlled Infusion in Emergency Department Sedation (ProTEDS)—a multi-centre feasibility study protocol
Source: Pilot Feasibility Stud. 2019 Feb 18;5:27. doi: 10.1186/s40814-019-0412-y (PMC6378735; doi:10.1186/s40814-019-0412-y)
Supplement: Supplementary file 1 — Patient satisfaction—visual analogue scale (VAS) (PDF 30 kb) [file 40814_2019_412_MOESM1_ESM.pdf]

How satisfied were you with the procedure? Please mark on the line below with a vertical mark.

Not satisfied | Very satisfied
