# Supplementary material for: A study protocol for a feasibility study: Propofol Target-Controlled Infusion in Emergency Department Sedation (ProTEDS)—a multi-centre feasibility study protocol
Source: Pilot Feasibility Stud. 2019 Feb 18;5:27. doi: 10.1186/s40814-019-0412-y (PMC6378735; doi:10.1186/s40814-019-0412-y)
Supplement: Supplementary file 4 — The TCI sedation flow sheet (PDF 71 kb) [file 40814_2019_412_MOESM4_ESM.pdf]

Apply patient monitoring and secure suitable IV access

Connect patient to the TCI pump and commence supplemental O_2 at 4 L min^{-1}

INITIAL PHASE: Set $\mathbf{C_{pT}}$ at 1.4µg ml^{-1} and Start TCI propofol (1%). Monitor $\mathbf{C_{eT}}$ and assess sedation as $\mathbf{C_{eT}}$ rises to 1.0 µg ml^{-1}, 1.2µg $ml^{-1}$and 1.4µg ml^{-1}. The patient is likely to be under sedated in this initial phase but follow the algorithm below if they become adequately sedated or require no further sedation.

If the patient remains under sedated when $\mathbf{C_{pT}}$ and $\mathbf{C_{eT}}$ **=** 1.4µg ml^{-1} then proceed to the **TITRATION PHASE.**

Adequately Sedated

OAA/S 3 (Response only after name is called loudly and/or repeatedly)

Do not change the pump settings

Under sedated

OAA/S 5 (Responds readily to name spoken in normal tone) **OR**

OAA/S 4 (Lethargic response to name spoken in normal tone)

Requires no further sedation

OAA/S 2 (Response only after mild prodding or shaking) **OR**

OAA/S 1 (Response only after painful trapezius squeeze) **OR**

OAA/S 0 (No response after painful trapezius squeeze)

Stop infusion and DO NOT give alfentanil bolus

TITRATION PHASE: If the patient remains under sedated when $\mathbf{C_{pT}}$ and $\mathbf{C_{eT}}$ **=** 1.4µg ml^{-1} then increase the $\mathbf{C_{pT}}$ by 0.2µg ml^{-1} every 2 minutes to a maximum of 4.0 µg ml^{-1}.

Asses the sedation level every 2 minutes. When $\mathbf{C_{pT}}$ is 2.6µg ml^{-1} then a second healthcare professional should also assess the sedation level before proceeding further to a maximum of 4.0 µg ml^{-1}.

If remains **OAA/S 5 OR OAA/S 4** at $\mathbf{C_{pT}}$ **= 4µg** $\mathbf{ml^{-1}}$ **then STOP STUDY AT THIS POINT**

Administer Alfentanil Bolus

>70kg give 250 µg

50-70kg give 200 µg

COMMENCE REDUCTION

On completion of the reduction the infusion should be stopped and the patient observed until complete recovery

If an adverse event occurs, the TCI should be stopped immediately
