# Supplementary material for: A study protocol for a feasibility study: Propofol Target-Controlled Infusion in Emergency Department Sedation (ProTEDS)—a multi-centre feasibility study protocol
Source: Pilot Feasibility Stud. 2019 Feb 18;5:27. doi: 10.1186/s40814-019-0412-y (PMC6378735; doi:10.1186/s40814-019-0412-y)
Supplement: Supplementary file 5 — A patient’s modified observer’s assessment of alertness/sedation score (PDF 37 kb) [file 40814_2019_412_MOESM5_ESM.pdf]

5. Responds readily to name spoken in normal tone

4. Lethargic response to name spoken in normal tone

3. Response only after name is called loudly and/or repeatedly

2. Response only after mild prodding or shaking

1. Response only after painful trapezius squeeze

0. No response after painful trapezius squeeze
